# International consensus statement regarding the use of animal models for research on anastomoses in the lower gastrointestinal tract

**DOI:** 10.1007/s00384-016-2550-5

**Published:** 2016-03-10

**Authors:** Joanna W. A. M. Bosmans, Martine Moossdorff, Mahdi Al-Taher, Lotte van Beek, Joep P. M. Derikx, Nicole D. Bouvy

**Affiliations:** Department of General Surgery, Maastricht University Medical Centre, P.O. Box 5800, 6202 AZ Maastricht, The Netherlands; NUTRIM School of Nutrition and Translational Research in Metabolism, Maastricht University, Maastricht, The Netherlands; GROW School for Oncology and Developmental Biology, Maastricht University, Maastricht, The Netherlands; Pediatric Surgical Center of Amsterdam, Emma Children’s Hospital University Medical Centre and VU Medical Centre, Amsterdam, The Netherlands

**Keywords:** Anastomotic healing, Consensus, Animal models, Experimental studies, Colorectal anastomosis

## Abstract

**Purpose:**

This project aimed to reach consensus on the most appropriate animal models and outcome measures in research on anastomoses in the lower gastrointestinal tract (GIT). The physiology of anastomotic healing remains an important research topic in gastrointestinal surgery. Recent results from experimental studies are limited with regard to comparability and clinical translation.

**Methods:**

PubMed and EMBASE were searched for experimental studies investigating anastomotic healing in the lower GIT published between January 1, 2000 and December 31, 2014 to assess currently used models. All corresponding authors were invited for a Delphi-based analysis that consisted of two online survey rounds followed by a final online recommendation survey to reach consensus on the discussed topics.

**Results:**

Two hundred seventy-seven original articles were retrieved and 167 articles were included in the systematic review. Mice, rats, rabbits, pigs, and dogs are currently being used as animal models, with a large variety in surgical techniques and outcome measures. Forty-four corresponding authors participated in the Delphi analysis. In the first two rounds, 39/44 and 35/39 participants completed the survey. In the final meeting, 35 experts reached consensus on 76/122 items in six categories. Mouse, rat, and pig are considered appropriate animal models; rabbit and dog should be abandoned in research regarding bowel anastomoses. ARRIVE guidelines should be followed more strictly.

**Conclusions:**

Consensus was reached on several recommendations for the use of animal models and outcome measurements in research on anastomoses of the lower GIT. Future research should take these suggestions into account to facilitate comparison and clinical translation of results.

**Electronic supplementary material:**

The online version of this article (doi:10.1007/s00384-016-2550-5) contains supplementary material, which is available to authorized users.

## Introduction

Anastomotic leakage (AL) is one of the most dreaded complications after colorectal surgery and leads to high morbidity and mortality [1, 2]. Despite accumulated knowledge, improvement of surgical techniques, and ongoing research on this topic, the incidence of colorectal AL remains approximately 11 % worldwide [3].

Animal models are used on a regular basis to investigate normal healing of an intestinal anastomosis as well as leakage of the anastomosis. These animal models are vital for our understanding of anastomotic healing and introduction of new therapies for reduction of AL. However, over the past decades, a variety of animal models have been used which leads to heterogeneity, accompanied by differing anatomy and physiology between species. Recently, a systematic review concluded that animal research on AL is of poor quality and improvement is needed before results can be translated into the human setting [4].

In addition to the variety of animal models, a wide range of study endpoints and/or goals is used. The majority of studies examined the effect of a certain intervention on anastomotic healing, for example, aiming at improving anastomotic strength or reducing leakage rate in models of insufficient anastomoses. Several studies have focused on different techniques to perform the anastomosis, for example, evaluating or enforcing suturing techniques or various types of staplers [5–10]. Glues and patches have also been used to cover the anastomosis in an attempt to decrease leakage rate [11–19]. Other methods include stenting the lumen of the intestine or providing specific medication to improve wound healing [20–28]. The influence of specific drugs that may attenuate the anastomotic healing process was also investigated [29–32]. Due to the large variance in study design, outcome measures, and analyses for surrogate markers of anastomotic healing, the question arises whether there is one specific animal model suitable to investigate all these different research topics. Furthermore, the role of limitations such as availability, costs, handling, and housing requirements in choosing an animal model remains unclear and may also influence why certain animal models are currently being used.

To date, a single recommendation on the use of animal models for clinical colon AL has been proposed by Pommergaard et al. in 2011 [33]. Based on a systematic review, the authors first listed pros and cons for different experimental animals and subsequently opted for the mouse as best suited to mimic clinical colon AL. However, despite this recommendation, the use of mice to study bowel anastomoses has not been universally adopted. This suggests that there is either insufficient knowledge or limited support from researchers in the field. This lack of consensus, and the resulting inconsistencies and differences between reported research, limit transparency and opportunities to compare results between studies [4].

Ideally, the most suited model can be selected based on clear evidence regarding reproducibility and validity of a model. However, in animal research regarding bowel anastomoses, there is no single animal model that is evidently the most appropriate regarding practical ease, costs, reproducibility, and clinical translation. Therefore, expert consensus is a suitable method to achieve homogeneity in the selection of animal models. If consensus can be reached, there will be more support from fellow researchers leading to more frequent use of similar models. As a consequence, future research about anastomotic healing will become more comparable.

The aim of this study is to review different experimental studies in which an animal model was used to investigate either intestinal anastomotic healing or leakage and obtain information on the used animal models, location and type of surgery, macroscopic outcome, histological assessment, mechanical and biochemical outcome measures, and animal testing and welfare. Further, we aim to reach consensus on these subjects by performing a Delphi-based analysis using an online survey to collect judgements of animal surgeons who performed the studies reviewed here.

## Methods

### Literature search and study selection

In December 2014, an extensive literature search was conducted using the PubMed (MEDLINE) OvidSP (EMBASE) databases for all papers related to animal models, which were used in an experimental setting to either investigate anastomotic healing or anastomotic leakage in the lower gastrointestinal tract. Search terms included: “anastomosis/anastomotic,” “leak/leakage,” “bowel/intestine/colon/colorectal,” and “animal/animals/rat/mouse/mice/pig/dog/goat/rabbit/animal model.” English and Dutch were used as language restrictions and the search was limited to articles published between January 1, 2000 and December 1, 2014. The following inclusion criteria were applied to the titles and abstracts of the search results: experimental setting, use of an animal model, and an anastomosis made in the lower gastrointestinal tract (GIT) (gastroduodenal/gastrojejunal were considered upper GIT and therefore excluded). We excluded commentary reports, review articles, and articles containing results that had been previously reported in another included article. All articles were combined in a single list of which JWAMB and LvB identified eligible reports; in case of discrepancy, agreement on inclusion was reached through discussion with MA-T as a third reviewer. For an overview of the study selection, performed according to the Preferred Reporting Items for Systematic Reviews and Meta-Analyses (PRISMA) guideline, see supplementary data figure S1.

### Study outcomes

Our primary outcomes were (1) type of animal used and (2) location and type of anastomosis. As secondary outcomes, we evaluated scoring models used for macroscopic findings reporting on AL, i.e., adhesions, bursting pressure, histology, and other examinations performed. Further, we assessed the country of origin where the study was performed and the year in which the study was published and how animal welfare was reported.

### Online survey—adaptation from the Delphi technique

The qualitative review of the literature served as our starting point for the Delphi technique. The main goal was to achieve consensus on the use of animal models for research on anastomoses in the lower gastrointestinal tract, specifically on which animal, location, and type of surgery; macroscopic outcome; histological assessment; mechanical and biochemical outcome measures; and animal testing and welfare. The Delphi technique is a widely used and accepted consensus method for gathering data from respondents within their domain of expertise in order to formulate recommendations or guidelines that can be used in the future. For this report, we contacted all principal investigators from included articles by email, explained our study, and invited them to participate in this international Delphi project.

Questionnaires were developed and distributed using SurveyMonkey (SurveyMonkey Inc, Palo Alto, CA; www.survey-monkey.com). This online survey contained several questions on three main subtopics of intestinal anastomotic research: the first part consisted of questions regarding animal model used and reasoning for the choice of this model; the second part focused on macroscopically scoring and measurements performed on the anastomosis (leakage rate, adhesion evaluation), and the last part inquired about histological analysis and additional tests (e.g., bursting pressure, ELISA, quantitative polymerase chain reaction (qPCR)) that were used to gain more insight in the healing process (inflammation, proliferation). There were no open questions in the survey, but participants were encouraged to provide arguments for their choice, suggestions, or additional remarks in free text fields below each question.

After receiving participants’ responses, the collected information was converted into a second questionnaire. This round included the items and ratings summarized from the previous round. Here, we asked all participants to revise their judgments or specify reasons why they were not convinced of the most commonly used animal model. All items achieving consensus, remaining items, and their ratings, as well as minority opinions, were reported during the questionnaires. The RAND/UCLA Appropriate Method (RAM) [34] was used to assess consensus in an expert panel on the use of animal models, macroscopic scoring of leakage and adhesion, mechanical and biochemical parameters, and histological outcome. For reporting our study, we used the recommendations of Sinha et al. regarding the Delphi technique [35].

### Statistical analysis

Results of the survey were exported to MS Excel 2011 (Microsoft Corp, Redmond WA). Consensus was reached if the panel rated the subject inappropriate (panel median 1–3) or appropriate (panel median 7–9) on a 9-point scale without disagreement, according to the method used by Moossdorff et al. [36]. Disagreement was tested using the interpercentile range adjusted for symmetry in accordance to the RAND/UCLA Appropriateness Method Manual [34].

## Results

### Literature findings

**Fig. 1:**
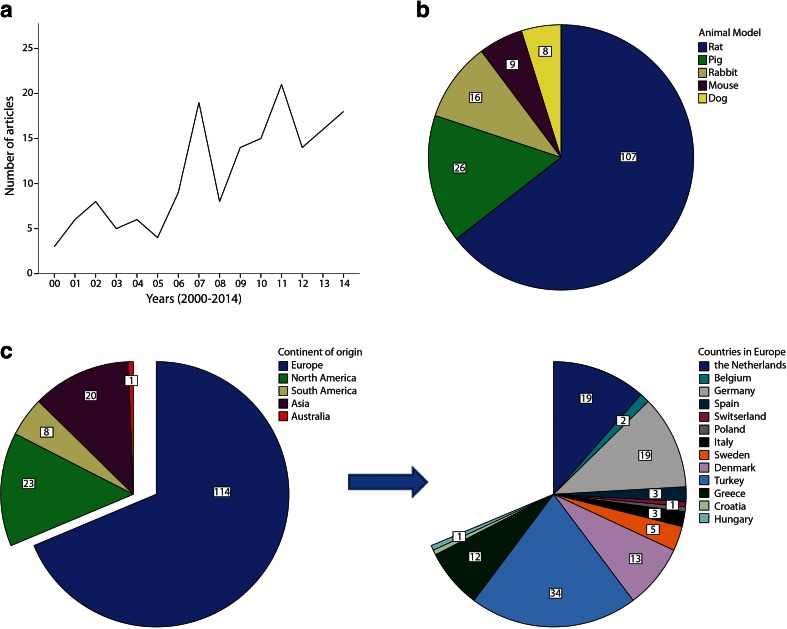
Incidence of articles published over the past 14 years (**a**). Animal models used in the studies (**b**) and origin of published articles (**c**)

In total, 277 articles were retrieved from the search. After screening, 167 articles were included in the systematic review (for flowchart see supplementary data S1). An ongoing increase in publication frequency on anastomotic healing and leakage was found, from only 3 in 2000 to 18 in 2014 (Fig. 1a). Animal models used in these experiments were rat (65 %), pig (15 %), rabbit (10 %), mouse (5 %), and dog (5 %) (Fig. 1b). From all 167 studies, only 4 reported laparoscopic surgery in animals, 3 in pig models [10, 37, 38], and 1 in a rat model [39]. One study performed transanal endoscopy [40], while all other studies used laparotomy. Research was mainly performed in Europe, with several research groups responsible for a relatively large contribution to the total number of articles (Fig. 1c).

Outcome measures reported in the included studies were anastomotic healing, anastomotic leakage, bursting pressure, tensile strength, adhesion scores, and histological parameters such as influx of granulocytes and collagen deposition. We summarized the characteristics of the used interventions and outcome measures that were used in these studies. This summary was sent to panel members as background information when completing the questionnaires. (PDF of the used questions during the Delphi rounds can be found online as supplementary data, S2, S3, and S4).

### Participation

**Fig. 2:**
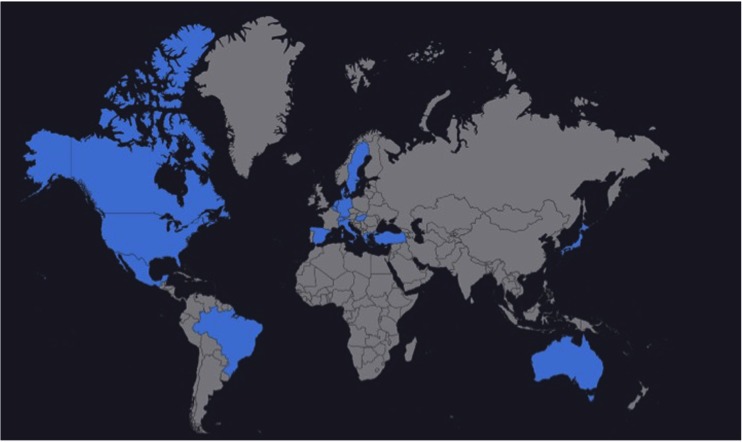
Participants in this Delphi analysis were from the main research groups that have published work on the subject of bowel anastomoses in animals, results obtained during the first survey (adapted from amcharts.com)

In total, 44 authors were willing to participate in the Delphi analysis, together being responsible for 77 of the included articles from the major research groups worldwide (Fig. 2). The first questionnaire was completed by 39/44 responders (89 % response rate). After non-responders were excluded, the second round was completed by 35/39 panel members, all of whom also completed the final round (Table 1). No additional people were invited as the Delphi progressed.

**Table 1:** Panel members that completed the entire Delphi analysis

Panel members	Institute
Magnus Ågren	University of Copenhagen, Denmark
John Alverdy	University of Chicago Medical Center, Chicago, IL, USA
Marcel Binnebösel	University Hospital of the RWTH, Aachen, Germany
Wim Ceelen	Ghent University Hospital, Belgium
Kadir Cetinkaya	Ankara Oncology Education and Research Hospital, Turkey
Jorge Cueto	Anahuac University, Mexico
Freek Daams	VU University Medical Center, The Netherlands
Alyssa Fajardo	Washington University, St. Louis, MO, USA
Laura Fresno	Autonomous University of Barcelona, Spain
Wolfgang Gaertner	University of Minnesota, Minneapolis, MN, USA
Torben Glatz	University Hospital Freiburg, Germany
Jens Höppner	University Hospital Freiburg, Germany
Niels Komen	University Hospital Leuven, Belgium
Guy Maddern	University of Adelaide, Australia
Antonio Morandeira-Rivas	“La Mancha Centro” General Hospital, Spain
Tyge Nordentoft	University of Copenhagen, Denmark
Adrian Park	Johns Hopkins University School of Medicine, Maryland, MD, USA
Pablo Parra Membrives	Valme University Hospital, Seville, Spain
Rogério Parra	University of São Paulo, Brazil
Troy Perry	University Medical Center Alberta, Canada
Hans-Christian Pommergaard	University of Copenhagen, Denmark
Manousos-Georgios Pramateftakis	Aristotle University of Thessaloniki, Greece
Dimitrios Raptis	Aristotle University of Thessaloniki, Greece, and Friedrich-Alexander University of Erlangen, Germany
Francisco Sánchez-De Pedro	“La Mancha Centro” General Hospital, Spain
Marc Schreinemacher	Maastricht University Medical Center, The Netherlands
Baddr Shakhsheer	University of Chicago Medical Center, Chicago, IL, USA
Juliette Slieker	CHUV University Hospital Lausanne, Switzerland
Lisette te Velde	VU University Medical Center, The Netherlands
Hideo Terashima	University of Tsukuba, Japan
Bobby Tingstedt	Lund University, Sweden
Go van Dam	University Medical Center Groningen, The Netherlands
Harry van Goor	Radboud University Medical Center, The Netherlands
Zhouqiao Wu	Erasmus University Medical Center, The Netherlands
Simon Yauw	Radboud University Medical Center, The Netherlands
Oded Zmora	Sheba Medical Center, Tel Aviv, Israel

### Characteristics of panel members

The occupation of the panel members is summarized in Table 2. Researchers had operated on approximately 200 animals and have published a median of four articles regarding this topic. All panel members were affiliated with a university and have experience performing animal research on this topic. The institutions listed in Table 1 were not involved in this project and do not necessarily subscribe to the consensus.

**Table 2:** Current functions of the panel members, results obtained during the first survey

Function	Number
Ph.D. Candidate	5
M.D./resident/surgeon	9
Postdoctoral researcher	4
Assistant Professor	11
Professor	10

All panel members have conducted hands-on animal experiments, mostly during their Ph.D. trajectory (17/35)

### First questionnaire

The first questionnaire consisted of 95 items in six categories, namely the choice of animal model, location and technique of the anastomosis, macroscopic outcome, histological assessment, mechanical and biochemical outcome measures, and reporting specifics on animal research. After the first round, consensus existed on 58 of the items (61 %) and disagreement or uncertainty on 37 items. Based on additional remarks, seven items were added and two were rephrased. The seven newly introduced items for the second round consisted of additional techniques in the category mechanical and biochemical outcome measures and a suggestion by one of the panel members for a new macroscopic scoring system.

### Second questionnaire

The second questionnaire was based on the first and consisted of 37 items on which consensus did not exist in the first round and 7 items added based on additional comments (total of 44 items). The most important item that was added in round 2 and on which immediate consensus was reached was the newly introduced Anastomotic Complication Score (ACS, see Table 3), which was proposed during the first round by one of the panel members. After the second round, consensus existed only on 3 items, in addition to the 58 items on which consensus was reached in the first round.

**Table 3:** Anastomotic complication score for macroscopic outcome in animal research regarding bowel anastomoses

	Anastomotic complication score
0	No adhesions or abnormalities
1	Adhesion to fat pad, clean anastomosis underneath
2	Adhesion to intestinal loop, abdominal wall or other organ
3	Anastomotic defect found underneath adhesion, no other abnormalities
4	Signs of possible contamination (e.g., small abscesses)
5	Clear anastomotic complication; spread of pus, obstruction at anastomosis, sign of peritonitis
6	Fecal peritonitis/Death due to peritonitis

### Final round

Feedback was provided to the participants after each round. In the final round, a clear distinction was made between positive and negative arguments from the second round to simulate a discussion between panel members. The topics for debate remained why certain animal models should or should not be used. Some panel members argued that small animal models are not able to reflect the clinical setting while others are certain that with the right scoring systems, one can obtain sufficient information to make the model more translational. Based on all arguments given by the panel members and the first two rounds, 20 recommendations were proposed. Consensus was reached on 17 of those recommendations (Table 4).

**Table 4:** Summary of the consensus on the use of animal models for bowel anastomoses in the lower gastrointestinal tract

Category	Consensus
Selection of animal model	- Mouse, rat and pig models are considered appropriate models
	- Choice of animal model depends on research question
	- A rat model is preferred to a mouse model (mostly because of size); however, knockout mice are helpful in answering specific research questions
	- Rabbit and dog models are not validated and are considered inappropriate to use
Location and type of surgery	- All locations in the colon (proximal, ascending, transverse, descending, sigmoid, and rectum) are considered appropriate
	- The small intestine should not be used for research purposes regarding anastomotic healing in the lower GIT
	- A resection is considered appropriate for constructing an anastomosis; no consensus was reached on using transection
	- Depending on the animal model, both open and laparoscopic surgery are considered appropriate
	- Interrupted sutures, running sutures (in all animals) or staplers (in the pig model) are considered appropriate to construct an anastomosis
Macroscopic outcome	- Anastomotic leakage should always be an outcome, preferably with different grades of leakage (small/large abscesses, fecal peritonitis, complete dehiscence)
	- The available scoring systems for grades of leakage were all considered inappropriate by the panel. The Anastomotic Complication Score may provide an appropriate scoring method for macroscopic outcome, but needs to be evaluated first
	- Adhesions to the anastomotic site are relevant as they might cover signs of leakage Adhesions in the abdominal cavity are less relevant and should only be taken into account in (anti)adhesion studies
Histological assessment	- Histological assessment is very valuable and considered as an appropriate outcome measure, especially in healing studies
	- Hematoxylin-eosine staining, Masson’s trichrome staining, and Picrosirius red staining are all considered appropriate for histological assessment
	- No specific histological score is considered appropriate for microscopic evaluation of the anastomosis; most important is the comparison with a control group
Mechanical and biochemical outcome measures	- Both bursting pressure and tensile strength are considered appropriate measurements for anastomotic strength. These measurements can be compared within one experiment, but due to heterogeneity not between different experiments - Additional outcome measures such as hydroxyproline content, amount of collagen, specific (immunohistochemistry) stainings, ELISA, and qPCR are not considered appropriate for specific anastomotic measurements but can provide information to answer specific research questions
Animal testing and welfare	- Blinding and randomization should be used and reported in animal studies
	- Detailed information on analgesia, anesthesia, antibiotics, antiseptic measures, intestinal segment involved, surgical technique, anastomotic complications as well as animal welfare is considered appropriate to report in studies. Many panel members suggested providing this as supplementary (online) data to the manuscript
	- ARRIVE guidelines are appropriate to follow and contribute to standardization [42]
	- An online registration of study protocols is considered appropriate for animal research

### Summary of items on which consensus was reached

In this study, consensus was reached among researchers studying intestinal anastomoses in animals. The main result is that the selection of an animal model depends on the research question and there is no “one size fits all.” Consensus was reached that mouse, rat, and pig models are considered appropriate models but dog and rabbit models should no longer be used for research on bowel anastomoses in the lower GIT.

The main outcome of the study (anastomotic healing/leakage) should always be evaluated macroscopically, where currently used scores were not considered appropriate enough. The Anastomotic Complication Score, as proposed by a panel member, may provide an objective scoring measure. Obviously, this new score needs to be evaluated in the experimental setting to obtain information about veracity and/or inter-observer variation, but it does seem to be a promising tool. Bursting pressure (or tensile strength) together with histological evaluation provides further information about the anastomosis. Additional analyses can be helpful to answer specific research questions but are not (yet) considered appropriate as surrogate markers for anastomotic healing. Reporting on animal testing and welfare is still not detailed enough in current literature and ARRIVE guidelines should be followed as much as possible.

A summary of items on which consensus was reached can be found in Table 4. A complete overview of the Delphi process with more detailed outcomes can be found online (S2, S3, and S4).

### Discussion points—items lacking consensus

The first topic of debate was the use of small animals. Many panel members felt that they were appropriate to use when systemic interventions are tested, but when interested in a local device, a larger animal is preferred. Although it is obvious to prefer to test devices intended for human use in an animal model of comparable size, testing a local device in a rat model is also considered acceptable. Mice should not be used to answer research questions on local devices. Another topic of debate was the use of mice and rats as models for healing or leakage. Many panel members felt that rats cannot be used as a model for AL since they are more resistant to infections and show hardly any clinical signs while other panel members have been using rats for this purpose for many years with very good results. Even though the rat is a validated model for both anastomotic healing and leakage, there are still opponents that claim that a rat is not suitable for this purpose, mostly based on own experiences. There was also disagreement on the consideration of practical ease in large animal models. Some believe that they are difficult in terms of anesthesia and housing, while others find them rather easy to handle and do not see any practical disadvantages.

For clinical translation to the human setting, all panel members agreed that the pig was the best-suited model in the preclinical setting. However, the use of mice as a clinically relevant model was also suggested, because it might mimic clinical AL better than the rat model. Despite solid arguments and a clinical scoring system proposed by one of the panel members, there was no consensus on the use of small animal models for clinical translation. The proposition to first test a hypothesis in a small animal model (mouse, rat, rabbit) and then use a large animal model (pig or dog) to make it more clinically transferable was not agreed upon and is therefore not recommended.

## Discussion

The frequency of studies that have used an animal model to investigate anastomotic leakage/healing in the lower gastrointestinal tract has increased considerably over the past decade, despite implementation of the 3R principle of Replacement, Reduction, and Refinement. As shown in a recent systematic review, reporting quality of these studies is poor and frequently insufficient [4]. Furthermore, a wide variability of animal models and measurement outcomes is used. This study aimed to reach consensus on the most appropriate animal models, outcome measures, and animal welfare in research on anastomoses in the lower gastrointestinal tract.

This project used the RAND/UCLA Appropriateness method (RAM) to develop consensus-based recommendation for the use of animal model in research on anastomoses in the lower gastrointestinal tract. While other methods can be used, the RAM is often used in biomedical research and considered as a solid method to use as it has strict parameters to what is defined as consensus.

All principal investigators of the studies included in the literature review were contacted, and the 35 panel members were responsible for 46 % of these studies. The authors that became panel members in this project were enthusiastic about the subject and working in one of the major research groups worldwide involved in experimental research regarding bowel anastomoses. Although this could have lead to selection bias, our approach is more objective than the “snowball method” in which experts are asked to provide email addresses of other experts. This method is also being used to form an expert panel in consensus studies [41]. Even though the number of panel members is an intrinsic limitation of any consensus project, we consider the panel to be a valid representation for researchers that perform animal research on bowel anastomoses in the lower gastrointestinal tract.

We invited authors from studies of the last decade, who are still publishing on this topic. It became clear that they sought to persuade other panel members with their arguments, especially at the beginning of the project. During the second round, all panel members were given their own answers in respect to the answers of the panel as well as arguments provided by other panel members. Obviously, all researchers are convinced of their own methods, believing that their models and techniques are best suited. However, during the project, panel members opened up for discussion, making it possible to indeed reach consensus and come up with suggestions and recommendations that can be a useful tool for directing future research.

In 2010, the NC3Rs Reporting Guidelines Working Group published the ARRIVE guidelines for reporting in vivo experiments in animals [42]. While considered appropriate to follow by the panel members in this study, few studies have actually used them to report animal research [43]. Panel members suggested that although these guidelines can contribute to an increase in standardization, and thus can be useful, they are also very detailed and complete. Most of the information required by the guidelines should be available in an online supplementary data section instead of in the manuscript. The panel also felt that it was appropriate to use an online registration for study protocols regarding animal research (comparable to clinicaltrial.gov in humans), which creates complete transparency. Moreover, they suggested standardized protocol online, per animal model, with guidelines to follow when performing animal research. More transparency in the methods would lead to refinement and reduction in animal experiments due to knowledge on teething problems experienced by others, leading to a decrease in learning time of each model. Also, innovative methods such as intestinal organoids or the use of human tissue that can replace animal models should be further investigated to reduce the use of animals, according to the 3R principle [44].

We recommend that future animal research that focuses on intestinal anastomosis should be conducted in a mouse, rat, or pig model and provide detailed information on analgesia, anesthesia, antibiotics, antiseptic measures, intestinal segment involved, surgical technique, anastomotic complications, as well as animal welfare. The ARRIVE guidelines should be followed more stringently to increase transparency in animal research. A publicly available online registry together with standardized protocols per animal model can aid in advancing the field of animal research on bowel anastomoses.

## Electronic Supplementary Material

Below is the link to the electronic supplementary material.

ESM S1 PRISMA flow chart of the review process. (DOC 27 kb)

ESM S2 Delphi round 1—questionnaire and appendix with additional information (PDF 758 kb)

ESM S3 Delphi round 2—questionnaire and discussion points from previous round (PDF 509 kb)

ESM S4 Delphi final consensus round and arguments provided by panel members (PDF 673 kb)
