# Supplementary material for: International consensus statement regarding the use of animal models for research on anastomoses in the lower gastrointestinal tract
Source: Int J Colorectal Dis. 2016 Mar 10;31:1021–30. doi: 10.1007/s00384-016-2550-5 (PMC4834109; doi:10.1007/s00384-016-2550-5)
Supplement: Supplementary file 1 — PRISMA flow chart of the review process. (DOC 27 kb) [file 384_2016_2550_MOESM1_ESM.doc]

**PRISMA flow chart of the review process**

## Records identified through PubMed and screened for retrieval

*n = 273*

Records after duplicates *(n = 28)* removed

*n* = 277

Articles screened

*n* = 277

Full text articles assessed for eligibility

*n* = 275

Studies included in qualitative review

*n* = 167

Articles excluded *n = 108*

# No English language n = 17 Upper GI/pancreatic duct n = 29 No anastomosis n = 30 Human studies n = 12 Other speciality n = 11 No in vivo study n = 9

# No full text available n = 2

## Records identified through EMBASE and screened for retrieval

*n = 32*
