# Supplementary material for: International consensus statement regarding the use of animal models for research on anastomoses in the lower gastrointestinal tract
Source: Int J Colorectal Dis. 2016 Mar 10;31:1021–30. doi: 10.1007/s00384-016-2550-5 (PMC4834109; doi:10.1007/s00384-016-2550-5)
Supplement: Supplementary file 2 — Delphi round 1—questionnaire and appendix with additional information (PDF 758 kb) [file 384_2016_2550_MOESM2_ESM.pdf]

Maastrict UMC+

azM | Maastricht University

The use of animal models for research on anastomoses in the lower gastrointestinal tract

Welcome to this Delphi survey!

Dear sir, madam,

Currently, we are performing a systematic review of all available literature on animal models concerning research on intestinal anastomoses in the lower gastrointestinal tract. Based on the data collected, we can conclude that there is no clear consensus about the animal models to be used, nor the outcomes to be measured. Since you have published one or more articles about this subject in the past years, we would like to ask your participation to complete this survey on the use of animal models for anastomotic healing/leakage. The Delphi method is a structured research technique, originally developed as a systematic, interactive forecasting method. The method relies on a panel of experts – like you. The experts answer questionnaires in two or more rounds. After each round, we provide an anonymous summary of the experts' answers from the previous round, as well as the reasons they provided for their judgments. Thus, experts are encouraged to revise their earlier answers in view of the replies of other members of their panel. It is believed that during this process the range of the answers will decrease and the group will converge towards a "consensus". Using this method, we hope to achieve a consensus on the use of intestinal anastomotic leakage models. Furthermore, we hope to formulate recommendations to structure the use of these models in the future.

We thank you for your participation.

Please do not hesitate to contact us if you have any more questions.

Nicole D. Bouvy MD PhD
Professor in innovative surgery
n.bouvy@mumc.nl

Anne-Claire (J.W.A.M.) Bosmans, MD
PhD candidate
ac.bosmans@maastrichtuniversity.nl

The use of animal models for research on anastomoses in the lower gastrointestinal tract

Example questions

This section gives an example of the type of questions asked in this survey. Please use the free text spaces to give arguments for your answers. These can be used in the following Delphi rounds to provide insight in the choices made by the expert panel.

Note: in case you are not sure about a certain topic or you feel that you don't have enough expertise to answer the question, please use 5 = not inappropriate/not appropriate.

1. *Example 1: How appropriate is it to include the following subjects asvegetables?
Please note that you will be asked the same question under "fruits".

	1: Very inappropriate	2	3	4	5	6	7	8	9: Very appropriate
Spinach	○	○	○	○	○	○	○	○	○
Cucumber	○	○	○	○	○	○	○	○	○
Potato	○	○	○	○	○	○	○	○	○
Tomato	○	○	○	○	○	○	○	○	○
Apple	○	○	○	○	○	○	○	○	○
Cauliflower	○	○	○	○	○	○	○	○	○

Additional remarks on why a certain subject should be considered a vegetable or other comments

2. *Example 2: How appropriate is it to include the following subjects as fruits?

	1: Very inappropriate	2	3	4	5	6	7	8	9: Very appropriate
Spinach	○	○	○	○	○	○	○	○	○
Cucumber	○	○	○	○	○	○	○	○	○
Potato	○	○	○	○	○	○	○	○	○
Tomato	○	○	○	○	○	○	○	○	○
Apple	○	○	○	○	○	○	○	○	○
Cauliflower	○	○	○	○	○	○	○	○	○

Additional remarks on why a certain subject should be considered a fruit or other comments

The use of animal models for research on anastomoses in the lower gastrointestinal tract

Choice of animal model

We saw in literature that the most used animal model for anastomotic healing was by far the rat (65%). Other animals used were pigs (15%), rabbits (10%), mice (5%) and dogs (5%). Use of the rat as an animal is practical, since the animals are easy to handle and not very expensive, however a rat might not represent a true clinical model due to its resistance to intra-abdominal infection. Therefore, it was suggested by Pommergaard et al that a mouse is a more clinical model for colorectal anastomotic leakage. Other authors suggest that the pig is most translational to the human setting, but pigs are expensive and not very easy to handle. The following questions regard the reasons why to choose a certain animal for research on anastomoses in the lower gastrointestinal tract.

3. Based on **costs**: How appropriate is the following model for research on anastomoses in the lower gastrointestinal tract?

	1: Very inappropriate	2	3	4	5	6	7	8	9: Very appropriate
Rat	○	○	○	○	○	○	○	○	○
Pig	○	○	○	○	○	○	○	○	○
Rabbit	○	○	○	○	○	○	○	○	○
Mouse	○	○	○	○	○	○	○	○	○
Dog	○	○	○	○	○	○	○	○	○

Please provide arguments for the animal model of your choice (rat, pig, rabbit, mouse or dog)/additional remarks

4. Based on practical ease: How appropriate is the following model for research on anastomoses in the lower gastrointestinal tract?

	1: Very inappropriate	2	3	4	5	6	7	8	9: Very inappropriate
Rat	○	○	○	○	○	○	○	○	○
Pig	○	○	○	○	○	○	○	○	○
Rabbit	○	○	○	○	○	○	○	○	○
Mouse	○	○	○	○	○	○	○	○	○
Dog	○	○	○	○	○	○	○	○	○

Please provide arguments for the animal model of your choice (rat, pig, rabbit, mouse or dog)/additional remarks

5. Based on clinical translation: How appropriate is the following model for research on anastomoses in the lower gastrointestinal tract?

	1: Very inappropriate	2	3	4	5	6	7	8	9: Very appropriate
Rat	○	○	○	○	○	○	○	○	○
Pig	○	○	○	○	○	○	○	○	○
Rabbit	○	○	○	○	○	○	○	○	○
Mouse	○	○	○	○	○	○	○	○	○
Dog	○	○	○	○	○	○	○	○	○

Please provide arguments for the animal model of your choice (rat, pig, rabbit, mouse or dog)/additional remarks

Research on intestinal anastomoses is carried out forseveral purposes: investigating the healing process, reducing anastomotic leakage with specific interventions or evaluation of new techniques.

6. Based on the purpose of observational research concerning anastomotic healing: How appropriate is the following model for research on anastomoses in the lower gastrointestinal tract?

	1: Very inappropriate	2	3	4	5	6	7	8	9: Very appropriate
Rat	○	○	○	○	○	○	○	○	○
Pig	○	○	○	○	○	○	○	○	○
Rabbit	○	○	○	○	○	○	○	○	○
Mouse	○	○	○	○	○	○	○	○	○
Dog	○	○	○	○	○	○	○	○	○

Please provide arguments for the animal model of your choice (rat, pig, rabbit, mouse or dog)/additional remarks

7. Based on the purpose to reduce anastomotic leakage with a systemic intervention: How appropriate is the following model for research on anastomoses in the lower gastrointestinal tract?

	1: Very inappropriate	2	3	4	5	6	7	8	9: Very appropriate
Rat	○	○	○	○	○	○	○	○	○
Pig	○	○	○	○	○	○	○	○	○
Rabbit	○	○	○	○	○	○	○	○	○
Mouse	○	○	○	○	○	○	○	○	○
Dog	○	○	○	○	○	○	○	○	○

Please provide arguments for the animal model of your choice (rat, pig, rabbit, mouse or dog)/additional remarks

8. Based on the purpose to reduce anastomotic leakage with a local intervention/device How appropriate is the following model for research on anastomoses in the lower gastrointestinal tract?

	1: Very inappropriate	2	3	4	5	6	7	8	9: Very appropriate
Rat	◯	◯	◯	◯	◯	◯	◯	◯	◯
Pig	◯	◯	◯	◯	◯	◯	◯	◯	◯
Rabbit	◯	◯	◯	◯	◯	◯	◯	◯	◯
Mouse	◯	◯	◯	◯	◯	◯	◯	◯	◯
Dog	◯	◯	◯	◯	◯	◯	◯	◯	◯

9. Overall, regarding all previous aspects, how appropriate is the following model for research on anastomoses in the lower gastrointestinal tract?

	1: Very inappropriate	2	3	4	5	6	7	8	9: Very appropriate
Rat	◯	◯	◯	◯	◯	◯	◯	◯	◯
Pig	◯	◯	◯	◯	◯	◯	◯	◯	◯
Rabbit	◯	◯	◯	◯	◯	◯	◯	◯	◯
Mouse	◯	◯	◯	◯	◯	◯	◯	◯	◯
Dog	◯	◯	◯	◯	◯	◯	◯	◯	◯

If you consider a different animal model as most appropriate, please specify and state reasons for your choice. You can also give arguments for the animal model of your choice (rat, pig, rabbit, mouse or dog).

Location in the lower gastrointestinal tract & type of surgery

It was evident that not all anastomoses were made in the same parts of the lower gastrointestinal tract. We excluded all anastomoses made from stomach-small intestine and anastomoses performed in the pancreatic-biliary tract. The most used anastomotic site was the colon (ascending 15%, transverse 20%, descending 32%, sigmoid 14%); however which part of the colon was not always evident (1% unknown). In addition, anastomoses were made in the small intestine (22%) and rectum (8%) as well. Some authors performed transection only, while others chose to resect a part of the bowel to reflect the human setting. Furthermore, laparoscopy was performed in a few cases, while the majority of the animal experiments was performed as open surgery.

10. How appropriate is it to perform an anastomosis in the following part to study anastomoses in the lower gastrointestinal tract?

	1: Very inappropriate	2	3	4	5	6	7	8	9: Very appropriate
Small intestine	○	○	○	○	○	○	○	○	○
Ascending colon	○	○	○	○	○	○	○	○	○
Transverse colon	○	○	○	○	○	○	○	○	○
Descending colon	○	○	○	○	○	○	○	○	○
Sigmoid	○	○	○	○	○	○	○	○	○
Rectum	○	○	○	○	○	○	○	○	○

Reasons for choosing a specific part of the intestine/additional remarks

11. How appropriate is it to perform an anastomosis in the following fashion to study anastomoses in the lower gastrointestinal tract?

	1: Very inappropriate	2	3	4	5	6	7	8	9: Very inappropriate
Laparoscopic	◯	◯	◯	◯	◯	◯	◯	◯	◯
Open	◯	◯	◯	◯	◯	◯	◯	◯	◯
Transection only	◯	◯	◯	◯	◯	◯	◯	◯	◯
With evident resection of the intestine	◯	◯	◯	◯	◯	◯	◯	◯	◯

Reasons to perform anastomotic surgery in a specific fashion/additional remarks

The use of animal models for research on anastomoses in the lower gastrointestinal tract

Macroscopic outcome

For outcome measures, the most abundant outcome measure was macroscopic observation of the anastomosis (intact, signs of leakage). The following questions will focus on macroscopic outcome measurements and scoring systems.

12. How appropriate is the following evaluation of the anastomosis in animal research on anastomoses in the lower gastrointestinal tract?

	1: Very inappropriate	2	3	4	5	6	7	8	9: Very appropriate
Macroscopic evaluation of the anastomotic site (anastomotic leakage or not)	○	○	○	○	○	○	○	○	○
A distinction between types of anastomotic leakage (e.g. abscess formation or anastomotic dehiscence)	○	○	○	○	○	○	○	○	○
A grading evaluation of the anastomosis (see appendix A)	○	○	○	○	○	○	○	○	○
Macrosopic evaluation of adhesions towards the anastomotic site	○	○	○	○	○	○	○	○	○
Microscopic evaluation of the anastomotic site (Wound healing assessment)	○	○	○	○	○	○	○	○	○

Why is a certain evaluation method considered appropriate/other important evaluations/additional remarks

13. Adhesions towards the anastomotic site are commonly observed. These adhesions can be scored regarding both quantitative and qualitative aspects of adhesions.How appropriate is it to use the following scoring system to score adhesions around the anastomosis in animal research on anastomoses in the lower gastrointestinal tract?

	1: Very inappropriate	2	3	4	5	6	7	8	9: Very appropriate
Adhesion scoring according to van der Ham et al (see appendix B)	○	○	○	○	○	○	○	○	○
Adhesion scoring according to Knightly et al (see appendix C)	○	○	○	○	○	○	○	○	○
Adhesion scoring according to Evans et al (see appendix D)	○	○	○	○	○	○	○	○	○
Adhesion scoring according to Bothin et al (see appendix E)	○	○	○	○	○	○	○	○	○
Adhesion scoring according to Zühlke et al (see appendix F)	○	○	○	○	○	○	○	○	○
Adhesion scoring according to Nair et al (see appendix G)	○	○	○	○	○	○	○	○	○
Adhesion scoring using a generic 0-3 scale (see appendix H)	○	○	○	○	○	○	○	○	○

Why is a certain scoring system considered appropriate/other adhesion scoring systems/additional remarks

Histological assessment

In order to define anastomotic healing, histological evaluation is often performed. The following question will regard different stainings and scoring mechanisms for this purpose.

14. How appropriate are the following stainings for histological evaluation of the anastomosis in animal research on anastomoses in the lower gastrointestinal tract?

	1: Very inappropriate	2	3	4	5	6	7	8	9: Very appropriate
Hematoxylin-eosin staining	○	○	○	○	○	○	○	○	○
Masson’s trichrome staining	○	○	○	○	○	○	○	○	○
Pico Sirius Red staining	○	○	○	○	○	○	○	○	○

Other important stainings or remarks

15. How appropriate is the use of the scoring system according to the following model (which can be found in the appendix) for <u>tissue evaluation of the anastomosis</u> in animal research on anastomoses in the lower gastrointestinal tract?

	1: Very inappropriate	2	3	4	5	6	7	8	9: Very appropriate
Ehrlich-Hunt model (see appendix I)	○	○	○	○	○	○	○	○	○
Ehrlich-Hunt model, modified by Philips et al (see appendix J)	○	○	○	○	○	○	○	○	○
Model by Verhofstad et al (see appendix K)	○	○	○	○	○	○	○	○	○
Model by Houdart et al & Hutschenreiter et al, modified by Garcia et al (see appendix L)	○	○	○	○	○	○	○	○	○
Generic histological scale for wound healing (see appendix M)	○	○	○	○	○	○	○	○	○

Why is a certain scoring system appropriate/other scoring systems/additional remarks

Mechanical & Biochemical outcome measures

In order to obtain more information on the anastomosis, additional tests are performed. For mechanical testing, the bursting pressure or tensile strength are often used. In early 2000s, measuring hydroxyproline content was the most performed biochemical assay while in later years measuring matrix metalloproteinase (MMP) activity was more often performed. The following question will focus on mechanical & biochemical outcome measures in animal research on anastomoses in the lower gastrointestinal tract.

16. How appropriate is it to use the following method as a measure for anastomotic healing in animal research on anastomoses in the lower gastrointestinal tract?

	1: Very inappropriate	2	3	4	5	6	7	8	9: Very appropriate
Bursting pressure	○	○	○	○	○	○	○	○	○
Tensile strength	○	○	○	○	○	○	○	○	○
Infusion with methylene-blue-stained saline	○	○	○	○	○	○	○	○	○
Radiological examination of leakage with contrast	○	○	○	○	○	○	○	○	○
Hydroxyproline content	○	○	○	○	○	○	○	○	○
Matrix metalloproteinase (MMP) activity	○	○	○	○	○	○	○	○	○
qPCR of bacterial 16S rDNA for bacterial translocation or microbial identification	○	○	○	○	○	○	○	○	○
Immunohistochemistry (please specify which stainings you consider appropriate)	○	○	○	○	○	○	○	○	○
ELISA on cytokines (please specify which cytokines you consider appropriate)	○	○	○	○	○	○	○	○	○
Other analyses (please specify which analyzing methods)	○	○	○	○	○	○	○	○	○

Please provide arguments on why you consider this appropriate or specify which method you used

Animal testing & welfare

The following section focuses on specific details of the experiments and animal welfare. Often, these items are not reported in scientific papers, while it can provide significant information. Using an anastomotic leakage model may cause severe discomfort for the animals and some researchers use this as an outcome measure. The following questions are about the appropriateness of using and reporting animal welfare assessments as well as reporting details about the experiments.

17. How appropriate is it to use & report the following items in studies on anastomoses in the lower gastrointestinal tract?

	1: Very inappropriate	2	3	4	5	6	7	8	9: Very appropriate
Approval of animal experiments (including protocol number)	○	○	○	○	○	○	○	○	○
Use of pain treatment & other medication	○	○	○	○	○	○	○	○	○
All materials used during the experiments (e.g. sutures)	○	○	○	○	○	○	○	○	○
Body weight of the animals	○	○	○	○	○	○	○	○	○
Reaching humane endpoints	○	○	○	○	○	○	○	○	○
Mortality of the animals	○	○	○	○	○	○	○	○	○
Morbidity of the animals (e.g. need for additional pain medication or complications)	○	○	○	○	○	○	○	○	○
An overview of animal welfare scores (e.g. ratings according to Komen et al for mice, appendix N or other generic scoring methods for animals, appendix O)	○	○	○	○	○	○	○	○	○

If you consider any other items appropriate regarding animal welfare, please specify/any additional remarks.

Maastricht UMC+

azM | Maastricht University

The use of animal models for research on anastomoses in the lower gastrointestinal tract

Researchers' characteristics

We are also interested in who performes animal research on anastomoses in the lower gastrointestinal tract. The following questions will focus on characteristics of researchers.

18. How many animals did you operate on?

- <10
- 11-30
- 31-50
- 51-100
- 101-200
- >200

19. How many articles did you publish on the subject of animal research on anastomoses in the lower gastrointestinal tract?

- 1
- 2-4
- 5-7
- 8-10
- >10

20. What was your function at the time of performing animal research on anastomoses in the lower gastrointestinal tract?

- Student
- PhD Candidate
- Postdoctoral Researcher
- Assistent Professor
- Professor
- Other, namely

21. What is your current function?

- Student
- PhD Candidate
- Postdoctoral Researcher
- Assistent Professor
- Professor
- Other, namely

Maastricht UMC+

azM | Maastricht University

The use of animal models for research on anastomoses in the lower gastrointestinal tract

Thank you very much!

We like to thank you for completing the first round of the Delphi survey. All responses will be analysed and the results will be reported back to you in the second survey. If you have any remarks at this point, or you want to add certain aspects about animal research on anastomoses in the lower gastrointestinal tract, please feel free to comment in the box below.

If you want direct contact through email, please send your message to:
n.bouvy@mumc.nl or ac.bosmans@maastrichtuniversity.nl

Thank you very much for participating in this international consensus project!

22. Additional remarks
