# Supplementary material for: International consensus statement regarding the use of animal models for research on anastomoses in the lower gastrointestinal tract
Source: Int J Colorectal Dis. 2016 Mar 10;31:1021–30. doi: 10.1007/s00384-016-2550-5 (PMC4834109; doi:10.1007/s00384-016-2550-5)
Supplement: Supplementary file 3 — Delphi round 2—questionnaire and discussion points from previous round (PDF 509 kb) [file 384_2016_2550_MOESM3_ESM.pdf]

Maastricht UMC+

azM | Maastricht University

Round 2: The use of animal models for research on anastomoses in the lower gastrointestinal tract

Welcome to the second round of this Delphi survey!

Dear panel members,

Congratulations on finishing the first round of our project towards consensus on animal research regarding anastomoses in the lower gastrointestinal tract.

It is a great pleasure to announce that we have already reached consensus on 61% of the items in the first questionnaire!

In this second round, we will build further on these results, to achieve consensus on the use of animal models, performing the anastomosis and additional analyses. The results of the first survey were analysed according to the RAND/UCLA Appropriateness Method Manual. If you want to receive more information on this process, please send us an email and we will provide more details.

For each item, we determined if the panel classified it as "inappropriate" [median 1-3], "uncertain" [median 4-6] or "appropriate" [median 7-9]. Next, we determined if you agreed sufficiently to rule out "disagreement". Consensus exists only on items rated as "appropriate" or "inappropriate" without disagreement.

If consensus did not exist, the question is repeated in this survey. To give an indication of the rating of your fellow panel members, the panel's median score on the 9 point scale, the range of the score and any additional remarks that you have entered are included in the question.
If questions were unclear or ambiguous, we adjusted them for clarification. This is noted in the question.

We will send your own answers to the first questionnaire to you by email, so you can check how your answers relate to the panel median. We hope this will help you decide whether or not you want to adjust your rating.

Completing the questionnaire should take about 15-20 minutes. As previously, you can quit the survey at any point and resume later by using the link in the email.

The deadline to this survey is Sunday March 1.

Please do not hesitate to contact us if you have any more questions.

Nicole D. Bouvy, MD PhD
Professor in innovative surgery

n.bouvy@mumc.nl

Anne-Claire (J.W.A.M.) Bosmans, MD
PhD candidate
ac.bosmans@maastrichtuniversity.nl

P.S. thank you very much for your feedback on some questions, particularly regarding the 9 point scale. We made sure that this is undoubtably clear in this round.

Round 2: The use of animal models for research on anastomoses in the lower gastrointestinal tract

Example questions; to include as fruit or vegetable

1. *Example 1: How appropriate is it to include the following subjects as vegetables?
Please note that you will be asked the same question under "fruits".

Consensus was reached on the following items:

Spinach: appropriate
Cauliflower: appropriate
Cucumber: appropriate
Potato: appropriate
Apple: inappropriate
Tomato: appropriate

Maastricht UMC+

azM | Maastricht University

Round 2: The use of animal models for research on anastomoses in the lower gastrointestinal tract

Choice of animal model

We saw in literature that the most used animal model for anastomotic healing was by far the rat (65%). Other animals used were pigs (15%), rabbits (10%), mice (5%) and dogs (5%). Use of the rat as an animal is practical, since the animals are easy to handle and not very expensive, however a rat might not represent a true clinical model due to its resistance to intra-abdominal infection. Therefore, it was suggested by Pommergaard et al that a mouse is a more clinical model for colorectal anastomotic leakage. Other authors suggest that the pig is most translational to the human setting, but pigs are expensive and not very easy to handle. The following questions regard the reasons why to choose a certain animal for research on anastomoses in the lower gastrointestinal tract.

* 2.

Based on **costs**, consensus was reached on the following items:

Rat: appropriate
Mouse: appropriate
Pig: inappropriate
Dog: inappropriate

Consensus was not reached on the **rabbit:** panel median 5 | Range 3-7

Additional remarks:
"Rabbit: in my opinion the most relevant in terms of clinical representation, costs and model."
"Rodents that can be housed in groups in cages need less costly facilities and mantainance than rabbits, and especially, dogs and pigs"
"Rabbit models have no real benefit over rat models"
"Rabbits are cheap"

Q: Based on costs: How appropriate is the rabbit model for research on anastomoses in the lower gastrointestinal tract?

	1: Very inappropriate	2	3	4	5	6	7	8	9: Very appropriate
Rabbit	○	○	○	○	○	○	○	○	○

Please provide arguments on why you feel this way about the rabbit or any other additional remarks

* 3.

Based on **practical ease**, consensus was reached on:

Rat: appropriate
Mouse: appropriate

No consensus was reached on the use of the other animals, based on practical ease.

Rabbit: panel median 6 | range 4.5-7.5
Dog: panel median 5 | range 3-6
Pig: panel median 5 | range 3-8

Additional remarks:
"Anesthesia is impractical in the rabbit"
"Anesthesia for rats and rabbits is quite simple and an assistant is not required for the intervention. Interventions can be done much quicker."
"Rabbits are easy to maintain and operations are easy to perform"
"Obviously, bigger animal models may ease suture and anastomosis performance. Rats and rabbits are still big enough to make surgery easy"
"Pigs provide a large animal model with a correspondingly large GI tract"
"Pigs can be used for feasibility studies, however, due to size and higher maintenance this model is not suited for studies requiring large numbers"
"The bigger animals require more preparation for a surgical intervention"
"Dog models have no real benefit over pig models"

Just to be clear; with practical ease, we mean not only the operation itself, but please also take into account the size of the animals (handling/manipulation), the use of anesthesia and housing.

Based on practical ease: How appropriate is the following model for research on anastomoses in the lower gastrointestinal tract?

	1: Very inappropriate	2	3	4	5	6	7	8	9: Very appropriate
Rabbit	○	○	○	○	○	○	○	○	○
Dog	○	○	○	○	○	○	○	○	○
Pig	○	○	○	○	○	○	○	○	○

Please provide arguments why you consider the rabbit, pig, or dog as (in)appropriate/additional remarks

* 4.

Consensus was reached on the following items based on clinical translation:
Pig: appropriate
Dog: appropriate

No consensus was reached for the smaller animals:
Rat: panel median 5 | range 4-7
Rabbit: panel median 5 | range 4.8-6.2
Mouse: panel median 6 | range 3.8-7.2

Additional remarks:
"In case of leakage, you need a model that gets peritonitis like humans"
"Rat is too resistant for infections"
"Rat model heals very fast, therefore it is considered one of anastomotic healing instead of leakage"
"A new interesting model by Wu et al has been developed in the rat. In this setup, a regular partial colectomy is performed, which produces leakage rates comparable to that of the pig and mouse model, the two animal models that are considered the best models for clinical leakage"
"The rat is considered the best-validated model for anastomotic healing due to the bulk of literature uses this animal. However, this may not necessarily indicate that the rat is superior, only that studies primarily have used this animal due to practical reasons. Moreover, relevant outcomes such as bursting pressure, breaking strength and histopathological scales have primarily been developed/validated in the rat"
"Mouse models should also be regarded as important, because most useful antibodies for FACS and other appropriate analysis methods are just available for mice"
"With response to intra-abdominal infection, I believe that the mouse is comparable to humans"
"Genetic manipulation in mice permits the study of specific pathways and genes involved in intestinal healing, moreover there is a large variety of reagents (f.e. antibodies) available for mouse studies"
"The use of knockout mice is very important"
"We must also consider if we can use the same materials and devices in human clinical practice with the model chosen"
"Remember, animal models do not need to be clinically relevant, they just need to be useful"

Based on clinical translation: How appropriate is the following model for research on anastomoses in the lower gastrointestinal tract?

	1: Very inappropriate	2	3	4	5	6	7	8	9: Very appropriate
Rat	○	○	○	○	○	○	○	○	○
Rabbit	○	○	○	○	○	○	○	○	○
Mouse	○	○	○	○	○	○	○	○	○

Please provide arguments for the animal model of your choice (rat, rabbit, mouse)/additional remarks

Research on intestinal anastomoses is carried out for several purposes: investigating the healing process, reducing anastomotic leakage with specific interventions or evaluation of new techniques.

* 5.

Based on the purpose of observational research concerning anastomotic healing, consensus was reached on the following animals being appropriate models: Rat, Pig, Dog, Mouse.
No consensus was reached on the rabbit:

Rabbit: panel median 5 | range 2-7

Additional remarks:
"Never worked with rabbits"
"All of them can be useful; their usefulness depends among other things on the type of intervention to be studied"
"Mechanism of anastomotic healing can be studied on all animals"
"No experience with rabbits"
"All of these models will be useful"

Based on the purpose of observational research concerning anastomotic healing: How appropriate is the following model for research on anastomoses in the lower gastrointestinal tract?

	1: Very inappropriate	2	3	4	5	6	7	8	9: Very appropriate
Rabbit	○	○	○	○	○	○	○	○	○

Additional remarks

* 6.

Based on the purpose to reduce anastomotic leakage with a systemic intervention, consensus was reached on the following animals:

Rat: appropriate
Pig: appropriate
Mouse: appropriate

No consensus was reached on rabbits and dogs:
Rabbit: panel median 5 | range 2-7
Dog: panel median 6.5 | range 5-8

Additional remarks:
"The larger the animals, the more it costs (forpharmaceuticals). Mouse and rabbit are easier to test different substances on in smaller quantities"
"Systematic interventions generally can be easy in any animal model"
"*Small animals are easier in handling and lower costs to facilitate the study of systemic interventions, even more so when the sample size required for the experiment is large"*
"Surgery does not affect rodents in the same way than pigs or dogs, this may introduce bias in the evaluation of the effect of the intervention that is being proved"
"Never worked with rabbits or dogs"

Based on the purpose to reduce anastomotic leakage with a systemic interventior How appropriate is the following model for research on anastomoses in the lower gastrointestinal tract?

	1: Very inappropriate	2	3	4	5	6	7	8	9: Very appropriate
Rabbit	○	○	○	○	○	○	○	○	○
Dog	○	○	○	○	○	○	○	○	○

Please provide arguments for the animal model of your choice (rabbit,dog)/additional remarks

* 7.

Based on the purpose to reduce anastomotic leakage with a local intervention/device, consensus was reached on the following items:
Pig: appropriate
Dog: appropriate

No consensus was reached on the following animals:
Rat: panel median 5.5 | range 3-6
Mouse: panel median 4 | range 2-6
Rabbit: panel median 5 | range 4.8-7

Additional remarks:
"*Larger animals are needed for medical or surgical interventions"*
"Relevant outcomes such as bursting pressure, breaking strength and histopathological scales have primarily been developed/validated in the rat"
"Dogs and pigs could provide a better view and would be easier to handle as they are bigger in size. However, we did not remark any difficulty in assessing our measurements in a rat model"
"In cases of mechanical intervention methods, e.g. rings, suture less anastomosis etc., a model with bigger animals could be more appropriate"

Based on the purpose to reduce anastomotic leakage with a local intervention/device How appropriate is the following model for research on anastomoses in the lower gastrointestinal tract?

	1: Very inappropriate	2	3	4	5	6	7	8	9: Very appropriate
Rat	○	○	○	○	○	○	○	○	○
Rabbit	○	○	○	○	○	○	○	○	○
Mouse	○	○	○	○	○	○	○	○	○

Additional remarks

* 8.

<u>Overall, regarding all previous aspects,</u> consensus was reached on the following animals:

Rat: appropriate
Pig: appropriate

No consensus was reached on the following animals:

Mouse: panel median 6 | range 4.8-7.2
Rabbit: panel median 5 | range 5-7
Dog: panel median 5 | range 4.8-7

Additional remarks:
"The answer depends heavily on the question of the research"
"Mice are hard to handle when it comes to anastomotic surgery, you need a large amount of animal surgical experience"
"Mice are most appropriate due to the fact that you can use knockout mice to test specific pathways, use large sample sizes and there are many reagents available for further analyses"
"The rabbit is a hard animal to keep and to anesthetize in my experience"
"Pigs and dogs are similar size as humans and therefore considered appropriate models"
"*The dog raises ethical issues due to its domestication"*
"There is no 'one size fits all' model, and the most appropriate model depends on the study question. most models dealing with a device or anastomotic technique are more appropriate with large animals, and most models dealing with systemic interventions are more efficient with small animals"

<u>Overall, regarding all previous aspects,</u> how appropriate is the following model for research on anastomoses in the lower gastrointestinal tract?

	1: Very inappropriate	2	3	4	5	6	7	8	9: Very appropriate
Rabbit	○	○	○	○	○	○	○	○	○
Mouse	○	○	○	○	○	○	○	○	○
Dog	○	○	○	○	○	○	○	○	○

If you consider rabbit, mouse or dog (in)appropriate, please specify and state reasons for your choice.

Round 2: The use of animal models for research on anastomoses in the lower gastrointestinal tract

Location in the lower gastrointestinal tract & type of surgery

It was evident that not all anastomoses were made in the same parts of the lower gastrointestinal tract. We excluded all anastomoses made from stomach-small intestine and anastomoses performed in the pancreatic-biliary tract. The most used anastomotic site was the colon (ascending 15%, transverse 20%, descending 32%, sigmoid 14%); however which part of the colon was not always evident (1% unknown). In addition, anastomoses were made in the small intestine (22%) and rectum (8%) as well. Some authors performed transection only, while others chose to resect a part of the bowel to reflect the human setting. Furthermore, laparoscopy was performed in a few cases, while the majority of the animal experiments was performed as open surgery.

* 9. On the location of the anastomosis, consensus was reached on the following items:

Ascending colon: appropriate
Transverse colon: appropriate
Descending colon: appropriate
Sigmoid: appropriate
Rectum: appropriate

No consensus was reached on performing an anastomosis in the small intestine:
Small intestine: panel median 4 | range 2.8-7

Additional remarks:
"Small intestine is inappropriate as the clinical relevance is mainly within the large bowel - also due to its inherent physiological characteristics"
"The small intestine heals too easily in animals I have performed research on and is therefore not to be trusted to be translational to humans or colonic anastomoses"
"A small intestine anastomosis likely will not reflect lower GI healing because the immunobiology and blood supply are much different"
"Due to less contamination, the small intestine should be avoided"
"Depending on the research question, all can be appropriate"
"Evidence suggests that mechanisms of anastomotic healing are different in small and large bowel anastomoses"

How appropriate is it to <u>perform an anastomosis in the small intestine</u> to study anastomoses in the lower gastrointestinal tract?

	1: Very inappropriate	2	3	4	5	6	7	8	9: Very appropriate
Small intestine	○	○	○	○	○	○	○	○	○

Reasons for regarding the small intestine as (in)appropriate

* 10.

Consensus was reached that it is appropriate to perform an anastomosis in the following fashion:
Laparoscopic: appropriate
Open: appropriate
Resection: appropriate

No consensus was reached on performing a transection only:
Transection: panel median 6 | range 5-8

Additional remarks:
"Most lower gastrointestinal tract pathologies imply to remove the sick segment of the intestine. Thus, anastomosis of a evident resection of the intestine should be more appropriate in this case. Removement of the affected segment together with its respective vasculature is to be considered in resection and anastomosis, as well as healing between different parts of the lower intestinal tract"
"Resection of the intestine seems essential to more accurately reflect the true clinical context. The potential effects of disrupting blood and lymphatic drainage can only be appreciated via a true resection."
"In general, transection is not clinical relevant, but it is more standardized"
"Using transection only allows homogenizing treatment groups, elimination other technical factors as impairment of blood supply or excessive traction on the area of the anastomosis"
"Transection only is sufficient in the mouse and pig"
"Again, it depends on the research question: 1) investigating healing, then transection might be good. 2) investigating leakage then resection is more relevant"
"An anastomosis is an anastomosis, with or without resection"

How appropriate is it to perform an anastomosis in the following fashion to study anastomoses in the lower gastrointestinal tract?

	1: Very inappropriate	2	3	4	5	6	7	8	9: Very appropriate
Transection only	○	○	○	○	○	○	○	○	○

Reasons to find transection only (in)appropriate

Round 2: The use of animal models for research on anastomoses in the lower gastrointestinal tract

Macroscopic outcome

For outcome measures, the most abundant outcome measure was macroscopic observation of the anastomosis (intact, signs of leakage). The following questions will focus on macroscopic outcome measurements and scoring systems.

11. Consensus was reached on all evaluation methods:

Macroscopic evaluation of the anastomotic site (anastomotic leakage or not): appropriate
A distinction between types of anastomotic leakage (e.g. abscess formation or anastomotic dehiscence): appropriate
A grading evaluation of the anastomosis (see appendix A): appropriate
Macrosopic evaluation of adhesions towards the anastomotic site: appropriate
Microscopic evaluation of the anastomotic site (Wound healing assessment): appropriate

* 12.

Adhesions towards the anastomotic site can be scored regarding both quantitative and qualitative aspects of adhesions. There was no consensus on any of the adhesion scoring scales:

van der Ham et al: panel median 6 | range 6-7.8
Knightly et al: panel median 6 | range 5-6
Evans et al: panel median 6 | range 5-7
Bothin et al: panel median 5 | range 4-7
Zuhlke et al: panel median 6 | range 5-7
Nair et al: panel median 5 | range 4-6
Generic scoring scale: panel median 6 | range 4.2-7

Additional remarks:
"Scoring according to appendix C to H are not merely related to anastomotic complication."
"The adhesion score in both anastomotic healing models and in adhesion models is just appropriate to quantitatively describe the amount of adhesion formation. More interesting is the quality of adhesion formation and therefore you need IHC or FACS analyses. Therefore it is both not helpful and necessary in anastomotic healing models."
"The more detailed the scoring system the better. Scoring system should involve both extent of adhesions and quality of present adhesions. Sometimes best to use two that combine these aspects."
"The quantity of adhesions do also reflect surgical trauma, and are not a good reflection of anastomotic healing"

"The type of grading system according to Bothin is a good system, but maybe some parameters regarding the quantity and quality of adhesions in this particular case are missing."
"In my opinion the qualitative aspects are most important to consider when evaluating the adhesions to the anastomosis. Simple scales are more easily applied and are more reproducible."
"The scale from van der Ham and eventually from Bothin would be feasible to be followed. The other scales are appropriate but a little bit confusing, f.e. there many subjective criteria (thickness of adhesion/easily separated). It depends on your intraoperative handling."
"The Evans scoring system is more likely to be reproducible."
"A good score has to reveal the pathological differences and should be easily applicable in most cases. The Zulke score and Evans score meets those criteria."
"No score is appropriate. It may be more important to give a practical and easily repeatable description of a score. Standardization is more important."
"Adhesions should be scored based upon how they can be transsected, blunt, sharp, traction etc. Models D (Evans) and F (Zulhke) meet this criterium. Models E (Bothin) is too complex.
"I have limited experience in the evaluation of the adhesion formation. I think that the scoring system according to Bothin is very comprehensive, but may be difficult the evaluate in practice, at least in the mouse. The scales by Knightly and Zühlke have a suitable detail level."
"Most of these scales have some subjective element assessing the severity of adhesions. Scale E (Bothin) is less subjective, at the cost of ignoring the severity of adhesions."
"My opinion is that depending on the investigator and their animal model, leaks should be defined by images. Each investigator should take a high definition picture of the gross anastomosis and make their own grade from 1-4"
"Perhaps 'leakage' and 'adhesion score' should be combined in one 'anastomotic complication scale, because tha

	1: Very inappropriate	2	3	4	5	6	7	8	9: Very appropriate
Adhesion scoring according to van der Ham et al (see appendix B)	○	○	○	○	○	○	○	○	○
Adhesion scoring according to Knightly et al (see appendix C)	○	○	○	○	○	○	○	○	○
Adhesion scoring according to Evans et al (see appendix D)	○	○	○	○	○	○	○	○	○
Adhesion scoring according to Bothin et al (see appendix E)	○	○	○	○	○	○	○	○	○
Adhesion scoring according to Zühlke et al (see appendix F)	○	○	○	○	○	○	○	○	○
Adhesion scoring according to Nair et al (see appendix G)	○	○	○	○	○	○	○	○	○

	1: Very inappropriate	2	3	4	5	6	7	8	9: Very appropriate
Adhesion scoring using a generic 0-3 scale (see appendix H)	○	○	○	○	○	○	○	○	○
New suggested scoring scale by one of the experts, combining 'leakage score' and 'adhesion score' (see below, Anastomotic Complication Scale, appendix P or below)	○	○	○	○	○	○	○	○	○

Why is a certain scoring system considered appropriate/other adhesion scoring systems/additional remarks

Tabel 1: Anastomotic Complication Score (New scoring system, suggested by one of the panel members)

	**Anastomotic Complication Score**
**0**	No adhesions or abnormalities
**1**	Adhesion to fatpad, clean anastomosis underneath
**2**	Adhesion to intestinal loop, abdominal wall or other organ
**3**	Anastomotic defect found underneath adhesion, no other abnormalities
**4**	Signs of possible contamination (e.g. small abcesses)
**5**	Clear anastomotic complication; spread of pus, obstruction at anastomosis, signs of peritonits
**6**	Fecal peritonitis/Death due to peritonitis

13. Consensus was reached on the following stainings for histological evaluation of the anastomosis in animal research on anastomoses in the lower gastrointestinal tract:

Hematoxylin-eosin staining: appropriate
Masson’s trichrome staining: appropriate
Pico Sirius Red staining: appropriate

Round 2: The use of animal models for research on anastomoses in the lower gastrointestinal tract

Histological assessment

In order to define anastomotic healing, histological evaluation is often performed. The following question will regard different stainings and scoring mechanisms for this purpose.

* 14. How appropriate is the use of the scoring system according to the following model (which can be found in the appendix) for tissue evaluation of the anastomosis in animal research on anastomoses in the lower gastrointestinal tract?

Consensus was reached on the Houdart & Hutschenreiter scale, modified by Garcia (appendix L), which was considered as appropriate.

Consensus was not reached on the following items:

Ehrlich-Hunt: panel median: 6 | range 5-7
Modified Ehrlich-Hunt: panel median 6 | range 5-7.8
Verhofstad: panel median 6 | range 5-7
Generic scale: panel median 5 | range 5-6

Additional remarks:
"A simple 3 point system can be applied to almost any parameter and is typically more reproducible than complex scores."
"*The Ehrlich and Hunt model as modified by Philips at al is the most appropriate, as it is detailed and worldwide accepted."*
"*These scoring systems seem to be especially qualitative, and a more quantitative method is demanded"*
"*The chosen system should not be too complicated. We still do not know the correlation of these findings to the clinical outcome. none of these models assess the width of the anastomotic wound which we find important"*
"*When a good pathologist is available, choosing any of these scores may be an appropriate option, otherwise it may be too difficult for most surgical researcher to investigate histology on his own."*
"*Many of these scores include evaluation of muscle continuations. We have concerns in this aspect, because histology usually only takes a very small part of the whole colon. It is certainly questionable whether this minimal part represents the whole colon regarding continuity"*
"*As long as a score is used, we lose information. the best option may be cell counting, an appropriate score consists both parameters of wound healing and anastomotic healing. Such a score may require expertise of pathologist."*
"*The Ehrlich-Hunt model is most appropriate, although less exhaustive regarding the cellular elements such as the degree of epithelization. However, it does consider aspects related to collagen deposition, which is important in the healing process"*

"*The use of a scoring system is important for uniformity of report and for statistical analysis. The fact that there are plenty of scales indicates that there are no widely agreed criteria for microscopic assessment of anastomotic healing."*

"All are biased and semi-quantitative"

"The problem of most of these scores is that hardly any conclusion can be based on them. In particular the E&H scores seem contradictory. For example, high scores are given for both prominent inflammatory cells and collagen deposition; the former may be a sign of poor healing, whereas the latter may indicate good healing."

	1: Very inappropriate	2	3	4	5	6	7	8	9: Very appropriate
Ehrlich-Hunt model (see appendix I)	○	○	○	○	○	○	○	○	○
Ehrlich-Hunt model, modified by Philips et al (see appendix J)	○	○	○	○	○	○	○	○	○
Model by Verhofstad et al (see appendix K)	○	○	○	○	○	○	○	○	○
Generic histological scale for wound healing (see appendix M)	○	○	○	○	○	○	○	○	○

Why is a certain scoring system appropriate/other scoring systems/additional remarks

Round 2: The use of animal models for research on anastomoses in the lower gastrointestinal tract

Mechanical & Biochemical outcome measures

In order to obtain more information on the anastomosis, additional tests are performed. For mechanical testing, the bursting pressure or tensile strength are often used. In early 2000s, measuring hydroxyproline content was the most performed biochemical assay while in later years measuring matrix metalloproteinase (MMP) activity was more often performed. The following question will focus on mechanical & biochemical outcome measures in animal research on anastomoses in the lower gastrointestinal tract.

* 15.

Consensus was reached on the following items as a appropriate measure for anastomotic healing:

Bursting pressure: appropriate
Tensile strength: appropriate

No consensus was reached on the use of the following items:

Methylene blue: panel median 6 | range 5-8
Radiological examination: panel median 6.5 | range 5-8
Hydroxyproline content: panel median 6 | range 5-7
MMP: panel median 6 | range 5-8
qPCR: panel median 5 | range 4-5.5
IHC: panel median 5 | range 4-6
ELISA: panel median 5 | range 3.9-5.1
Other: panel median 5 | range 4.8-6

How appropriate is it to use the following method as a measure for anastomotic healing in animal research on anastomoses in the lower gastrointestinal tract?

Additional remarks:
"Tissue is rapidly covered with methylene blue, which makes everything confusing, so I use saline alone instead"
"To determine presence of leakage only, methylene blue and radiological evaluation are both well suited, but not very practical"
"Radiological examination with contrast is practically inappropriate in small animals"
"Radiological examination is only practical in large animals"
"Both hydroxyproline and MMP activity can provide good outcomes at low costs"
"Analysis of hydroxyproline content allows conclusions on anastomotic healing"
"The technique of assessing hydroxproline is not easy and it is not rare to introduce methodological pitfalls,

same with MMP activity"
"Hydroxyproline content is less relevant, as it does not consider collagen types and mature/immature collagen and therefore not necessarily tissue strength."
"Combining MMP with bursting pressure is a proper evaluation of collagen formation and degradation"
"MMP is relevant to measure when evaluating interventions to inhibit collagen degradation"
"Using PCR is very sophisticated, however, perhaps a bit overshooting in an animal experimental setting"
"IHC and ELISA should include some general inflammatory markers"
"IHC may be useful by using antibodies for collagen, although it can be sometimes difficult to find specific antibodies in some species"
"In order to detect inflammatory cells like macrophages, lymphocytes etc. this should be done by FACS with specific antibodies"
"A good general cytokine that seems to be elevated with poor healing is TNF-alpha"
"Perhaps CRP-values are useful, because they are a relatively validated measure and also used in clinic to detect leakage"
"The problem we encountered was finding appropriate and valid antibodies for the animal species chosen"
"If bacterial translocation is being studied, FISH staining work well to identify bacteria within the tissues"
"New techniques to view real time healing would be appropriate"
"Measuring anastomotic viability might be a valuable tool to monitor the anastomosis, i.e. pO2, pH, pCO2"
"The clinical relevance of these surrogate markers is questionable"

	1: Very inappropriate	2	3	4	5	6	7	8	9: Very appropriate
Infusion with Methylene blue	○	○	○	○	○	○	○	○	○
Radiological examination of leakage with contrast	○	○	○	○	○	○	○	○	○
Hydroxyproline content	○	○	○	○	○	○	○	○	○
Matrix metalloproteinase (MMP) activity	○	○	○	○	○	○	○	○	○
qPCR of bacterial 16S rDNA for bacterial translocation or microbial identification	○	○	○	○	○	○	○	○	○
Immunohistochemistry (Ki67, neovascularisation, TUNEL)	○	○	○	○	○	○	○	○	○
ELISA on cytokines: mainly TNF-alpha, IL-6 & CRP (to a lesser extent: IL-1, IL-10, MCP-1)	○	○	○	○	○	○	○	○	○
FACS to detect inflammatory cells like macrophages and lymphocytes	○	○	○	○	○	○	○	○	○
FISH staining to identify bacterial translocation	○	○	○	○	○	○	○	○	○

	1: Very inappropriate	2	3	4	5	6	7	8	9: Very appropriate
Measuring anastomotic viability, with pO2, pH, pCO2	○	○	○	○	○	○	○	○	○
Imaging techniques, f.e. using MS-imaging (MALDI) or in situ hybridization (COX-2)	○	○	○	○	○	○	○	○	○
Electronic microscopy (TEM/SEM)	○	○	○	○	○	○	○	○	○
Microbiome studies	○	○	○	○	○	○	○	○	○
Collagen solubility using acetic acid/pepsin as a measure of collagen stability in anastomotic wounds	○	○	○	○	○	○	○	○	○

Please provide arguments on why you consider this appropriate or specify which method you used

Round 2: The use of animal models for research on anastomoses in the lower gastrointestinal tract

Animal testing & welfare

The following section focuses on specific details of the experiments and animal welfare. Often, these items are not reported in scientific papers, while it can provide significant information. Using an anastomotic leakage model may cause severe discomfort for the animals and some researchers use this as an outcome measure. The following questions are about the appropriateness of using and reporting animal welfare assessments as well as reporting details about the experiments.

16. Consensus was reached that the following items should be used & reported in studies on anastomoses in the lower gastrointestinal tract:

Approval of animal experiments (including protocol number): appropriate
Use of pain treatment & other medication: appropriate
All materials used during the experiments (e.g. sutures): appropriate
Body weight of the animals: appropriate
Reaching humane endpoints: appropriate
Mortality of the animals: appropriate
Morbidity of the animals (e.g. need for additional pain medication or complications): appropriate
An overview of animal welfare scores: appropriate

Even though consensus was reached on these topics, many panel members have noted that although the manuscript should focus on the research question. Detailed information on animals (antibiotics, randomization, blinding, antiseptic measures, diet) should be reported, but either in supplementary data (welfare scores in bundled form) or in an additional method section. It was also noted that the protocol numbers would be much more relevant is they were publicly available, cf. clinicaltrials.gov.

Maastricht UMC+

azM | Maastricht University

Round 2: The use of animal models for research on anastomoses in the lower gastrointestinal tract

Thank you very much!

We like to thank you for completing the second round of the Delphi survey. All responses will be analysed and the results will be reported back to you. After that, we will organize a conference call to aim at reaching more consensus and provide guidelines for animal research on anastomoses in the lower gastrointestinal tract.
If you have any remarks at this point, please feel free to comment in the box below.

If you want direct contact through email, please send your message to:
n.bouvy@mumc.nl or ac.bosmans@maastrichtuniversity.nl

Thank you very much for participating in this international consensus project!

17. Additional remarks
