# Supplementary material for: International consensus statement regarding the use of animal models for research on anastomoses in the lower gastrointestinal tract
Source: Int J Colorectal Dis. 2016 Mar 10;31:1021–30. doi: 10.1007/s00384-016-2550-5 (PMC4834109; doi:10.1007/s00384-016-2550-5)
Supplement: Supplementary file 4 — Delphi final consensus round and arguments provided by panel members (PDF 673 kb) [file 384_2016_2550_MOESM4_ESM.pdf]

Maastricht UMC+

azM | Maastricht University

# Final Round: The use of animal models for research on anastomoses in the lower gastrointestinal tract

## Welcome to the third round of this Delphi survey!

Dear panel members,

Congratulations on finishing the second round of our project towards consensus on animal research regarding anastomoses in the lower gastrointestinal tract.

It is a great pleasure to announce that we have reached consensus on 3 more items in the second questionnaire, the most important consensus was reached on the newly introduced Anastomotic Complication Score (ACS) that was considered appropriate for the evaluation of the anastomosis in animal research!

Please make sure that you read through all comments of the panel members which are send to you as a PDF file in a separate email, we only provide a few comments per item in this survey due to space limitation. The cases below are reflecting on the items that were scored ‘uncertain (5)’ in previous rounds. These items will not be repeated, but are revised in more general questions. Based on your previous answers, we made specific questions that enable us to formulate recommendations for animal research on bowel anastomoses in the lower GIT. Obviously, methods you have a lot of experience with are more likely to be scored as appropriate, but the aim of this final round should be to improve animal research on gastrointestinal anastomoses so please take the time to read all arguments before choosing your answer.

The deadline to this survey is Sunday May 3.

Please do not hesitate to contact us if you have any more questions.

Nicole D. Bouvy, MD PhD
Professor in innovative surgery
n.bouvy@mumc.nl

Anne-Claire (J.W.A.M.) Bosmans, MD
PhD candidate
ac.bosmans@maastrichtuniversity.nl

Maastricht UMC+

azM | Maastricht University

## Final Round: The use of animal models for research on anastomoses in the lower gastrointestinal tract

### Choice of animal model

**Conclusions so far:**

**· Mouse and rats are both considered to be cost-effective, so based on costs, consensus was reached that these models are appropriate to use. Also, there was consensus that pigs and dogs were inappropriate based on costs and no consensus was reached about the rabbit.**

**· Based on practical ease, consensus was reached that both the rat and the mouse are easy to handle. No consensus was reached on this topic for the other animals.**

**· Consensus was reached on both the pig and the dog being appropriate for clinical translation. No consensus could be reached on all smaller animals.**

**· Based on anastomotic healing research, all animals – except the rabbit – are considered appropriate models.**

**· To investigate the effect of a systemic intervention, consensus was reached that the rat, pig, dog and mouse all are appropriate models. The use of a rabbit model remained uncertain, probably due to less experience with this model**

**· Consensus was reached that both a pig and dog model are considered appropriate to perform research with if the aim is to reduce anastomotic leakage with a local intervention or device. On the use of small animals for this purpose, no consensus was reached.**

**· Overall, the rat and the pig are considered to be the most appropriate models for research on anastomoses in the lower gastrointestinal tract. No consensus was reached on the use of other animals such as rabbit, dog and mouse.**

* 1.

Many of the comments were that there is 'no one size fits all' and that 'choosing an animal depends on the research question' - see PDF for additional comments.

How appropriate is it to choose an animal model based on your research question (intervention, drug/biomaterial development, risk factors for healing)?

| 1: Very inappropriate | 2 | 3 | 4 | 5 | 6 | 7 | 8 | 9: Very appropriate |
|---|---|---|---|---|---|---|---|---|
| ○ | ○ | ○ | ○ | ○ | ○ | ○ | ○ | ○ |

Please provide arguments on why you feel this way or any other additional remarks

* 2. Comments given were: "Pigs and dogs obviously require greater intensity of care" and "Although large animals such as dog or pig facilitate surgical intervention, these are more cumbersome for handling, anesthesia and housing" - see PDF for additional comments.

Please note that with practical ease we mean not only the operation itself, but please also take into account the size of the animals (handling/manipulation), the use of anesthesia and housing.

How appropriate is it to use a large animal model (pig, dog) based on practical ease?

| 1: Very inappropriate | 2 | 3 | 4 | 5 | 6 | 7 | 8 | 9: Very appropriate |
|---|---|---|---|---|---|---|---|---|
| ○ | ○ | ○ | ○ | ○ | ○ | ○ | ○ | ○ |

Please provide arguments for your response/additional remarks

* 3.

Comments given: "Local interventions performed with autologous tissues or other biological samples can be challenging in small size animals" (con) & "Both mice and rat are very suitable" (pro) - see PDF for additional comments.

How appropriate is it to use a small animal model for the purpose of investigating the effect of a local intervention/device on anastomotic healing?

| 1: Very inappropriate | 2 | 3 | 4 | 5 | 6 | 7 | 8 | 9: Very appropriate |
|---|---|---|---|---|---|---|---|---|
| ○ | ○ | ○ | ○ | ○ | ○ | ○ | ○ | ○ |

Please provide arguments why you consider the small animals as (in)appropriate/additional remarks

* 4. "Rodents are inappropriate to translate the results to humans because of different diameters, intraluminal stools, microbioma etc." (con) & "Research in rodents can still be useful (pro), only there is a possible bias that we have to be honest about" - see PDF for additional comments.

How appropriate is it to use a small animal model (mouse, rat, rabbit) based on clinical translation?

| 1: Very inappropriate | 2 | 3 | 4 | 5 | 6 | 7 | 8 | 9: Very appropriate |
|---|---|---|---|---|---|---|---|---|
| ○ | ○ | ○ | ○ | ○ | ○ | ○ | ○ | ○ |

Please provide arguments for your response/additional remarks

* 5. "Devices that are developed for human use are different in size and difficult to test in rodents" & "For feasibility studies pigs and dogs are more suitable" - see PDF for additional comments.

How appropriate is it to first test your hypothesis in a small animal model (mouse, rat, rabbit) and then use a large animal model (pig, dog) to make it more clinically translational?

| 1: Very inappropriate | 2 | 3 | 4 | 5 | 6 | 7 | 8 | 9: Very appropriate |
|---|---|---|---|---|---|---|---|---|
| ○ | ○ | ○ | ○ | ○ | ○ | ○ | ○ | ○ |

Please provide arguments for your response/additional remarks

* 6. "The variety of knockout mice allows for the study of specific pathways in anastomotic healing, this seems translatable" & "Animal models need to be useful to advance the science and biology of the problem you are studying" - see PDF for additional comments.

How appropriate is the use of knockout mice to answer a specific research question?

| 1: Very inappropriate | 2 | 3 | 4 | 5 | 6 | 7 | 8 | 9: Very appropriate |
|---|---|---|---|---|---|---|---|---|
| ○ | ○ | ○ | ○ | ○ | ○ | ○ | ○ | ○ |

Please provide arguments for your response/additional remarks

* 7. "Working with rats is a bit easier compared to mice because of their size" & "The rat might be suited to evaluate improvement of healing with local intervention but not clinical leakage" - see PDF for additional comments.

How appropriate is it to <u>prefer a rat model to a mouse model</u> for animal research on bowel anastomoses in the lower GIT?

| 1: Very inappropriate | 2 | 3 | 4 | 5 | 6 | 7 | 8 | 9: Very appropriate |
|---|---|---|---|---|---|---|---|---|
| ○ | ○ | ○ | ○ | ○ | ○ | ○ | ○ | ○ |

Please provide arguments for your response/additional remarks

* 8. "Rabbits are more appropriate concerning the combination size-effectiveness" (pro) & "Rabbit are not suitable for postoperative analysis" (con) - see PDF for additional comments.

How appropriate is it to <u>use a rabbit model</u> in animal research on bowel anastomoses in the lower GIT if there is no consensus on the use of this animal model?

| 1: Very inappropriate | 2 | 3 | 4 | 5 | 6 | 7 | 8 | 9: Very appropriate |
|---|---|---|---|---|---|---|---|---|
| ○ | ○ | ○ | ○ | ○ | ○ | ○ | ○ | ○ |

Please provide arguments for your response/additional remarks

* 9. "Both rabbits and dogs could be used but they may not be the best model relative to other options" & "The dog and rabbit models need validation and should be abandoned until this validation takes place" - see PDF for additional comments.

How appropriate is it to <u>abandon the rabbit and the dog model</u> for animal research on bowel anastomoses?

| 1: Very inappropriate | 2 | 3 | 4 | 5 | 6 | 7 | 8 | 9: Very appropriate |
|---|---|---|---|---|---|---|---|---|
| ○ | ○ | ○ | ○ | ○ | ○ | ○ | ○ | ○ |

Please provide arguments for your response/additional remarks

* 10. "Basically we should support a small animal model and a big animal model for interventional and feasibility studies respectively" & "Rat model is best suited to study anastomotic healing, the mouse model for anastomotic leakage and the pig for feasibility" - see PDF for additional comments.

How appropriate is it to commit to three animal models (mouse, rat & pig) for animal research on bowel anastomoses?

| 1: Very inappropriate | 2 | 3 | 4 | 5 | 6 | 7 | 8 | 9: Very appropriate |
|---|---|---|---|---|---|---|---|---|
| ○ | ○ | ○ | ○ | ○ | ○ | ○ | ○ | ○ |

Please provide arguments for your response/additional remarks

Maastricht UMC+

azM | Maastricht University

## Final Round: The use of animal models for research on anastomoses in the lower gastrointestinal tract

### Location in the lower gastrointestinal tract & type of surgery

**· The small intestine is considered inappropriate as location of the anastomosis All participants considered the different locations in the large intestine as appropriate, so consensus was reached for all different parts of the colon.**
**· Both laparoscopic and open surgery were considered appropriate techniques for animal research.**
**· Consensus was reached thata resection is appropriate for the aim of anastomotic research, but no consensus was reached on using a transection only.**

* 11. How appropriate is it to use interrupted sutures for performing an anastomosis in animal research?

| 1: Very inappropriate | 2 | 3 | 4 | 5 | 6 | 7 | 8 | 9: Very appropriate |
|---|---|---|---|---|---|---|---|---|
| ○ | ○ | ○ | ○ | ○ | ○ | ○ | ○ | ○ |

Reasons for regarding this suture technique as (in)appropriate/additional remarks

* 12. How appropriate is it to use a running suture for performing an anastomosis in animal research?

| 1: Very inappropriate | 2 | 3 | 4 | 5 | 6 | 7 | 8 | 9: Very appropriate |
|---|---|---|---|---|---|---|---|---|
| ○ | ○ | ○ | ○ | ○ | ○ | ○ | ○ | ○ |

Reasons for regarding this suture technique as (in)appropriate/additional remarks

* 13. How appropriate is it to use a stapling device for performing an anastomosis in large animal models?

| 1: Very inappropriate | 2 | 3 | 4 | 5 | 6 | 7 | 8 | 9: Very appropriate |
|---|---|---|---|---|---|---|---|---|
| ○ | ○ | ○ | ○ | ○ | ○ | ○ | ○ | ○ |

Reasons for regarding this suture technique as (in)appropriate/additional remarks

## Final Round: The use of animal models for research on anastomoses in the lower gastrointestinal tract

### Evaluation of the anastomosis

**Conclusions so far:**

**· Consensus was reached on the following subjects regarding evaluation of the anastomosis: macroscopy, grading the anastomosis, typing the anastomosis, regarding adhesions towards the anastomotic site and microscopical evaluation. Participants find all types of evaluation appropriate in animal research on anastomoses.**

**· Consensus was reached on the newly introduced 'Anastomotic Complication Score' for the evaluation of the anastomosis.**

**· H&E, Sirius Red and Masson's trichrome staining were considered appropriate for histological evaluation and participants were most commonly experienced with H&E staining.**

**· No consensus was reached on using other techniques for the evaluation of the anastomosis, such as methylene blue, hydroxyproline, MMPs, qPCR and ELISA. Suggestions were made for several parameters by different experts, but on all suggestions no consensus was reached in Round 2.**

* 14. "It is not helpful and necessary to investigate adhesion formation in anastomotic healing models" & "scoring of adhesions to the anastomotic site is only useful when testing anti-adhesive material on anastomotic healing to unravel compartmentalization of anastomotic leak" - see PDF for additional comments

How appropriate is it to evaluate adhesions towards the anastomotic site?

| 1: Very inappropriate | 2 | 3 | 4 | 5 | 6 | 7 | 8 | 9: Very appropriate |
|---|---|---|---|---|---|---|---|---|
| ○ | ○ | ○ | ○ | ○ | ○ | ○ | ○ | ○ |

Why is it considered (in)appropriate/additional remarks

* 15. "It is not important which scoring system is used, it is important that there is a comparable control group" & "Scores don't lead to any usable interpretation or conclusion and are therefore not appropriate for classifying/measuring anastomotic healing" - see PDF for additional comments.

How appropriate is it to evaluate the anastomotic site with a histological scoring system?

| 1: Very inappropriate | 2 | 3 | 4 | 5 | 6 | 7 | 8 | 9: Very appropriate |
|---|---|---|---|---|---|---|---|---|
| ○ | ○ | ○ | ○ | ○ | ○ | ○ | ○ | ○ |

Why is it considered (in)appropriate/additional remarks

* 16. Since there was no consensus reached on any of the additional analytical methods in the previous rounds besides histology and bursting pressure/tensile strength, the following question arose:

How appropriate is it to perform additional analyses next to histology and bursting pressure/tensile strength?

| 1: Very inappropriate | 2 | 3 | 4 | 5 | 6 | 7 | 8 | 9: Very appropriate |
|---|---|---|---|---|---|---|---|---|
| ○ | ○ | ○ | ○ | ○ | ○ | ○ | ○ | ○ |

Why is it considered (in)appropriate/additional remarks

Maastricht UMC+

azM | Maastricht University

## Final Round: The use of animal models for research on anastomoses in the lower gastrointestinal tract

### Animal testing & welfare

**Consensus was reached that the following items are appropriate to use and report: AEC approval, pain treatment, materials used, reaching of humane endpoints, mortality and morbidity of the animals as well as animal welfare scores. Many participants gave suggestions on how to do this in practice, f.e. a supplementary method section or additional results on welfare score.**

* 17. How appropriate is it to follow the ARRIVE guidelines (see below) when reporting animal research?

| 1: Very inappropriate | 2 | 3 | 4 | 5 | 6 | 7 | 8 | 9: Very appropriate |
|---|---|---|---|---|---|---|---|---|
| ○ | ○ | ○ | ○ | ○ | ○ | ○ | ○ | ○ |

Why is it considered (in)appropriate/additional remarks

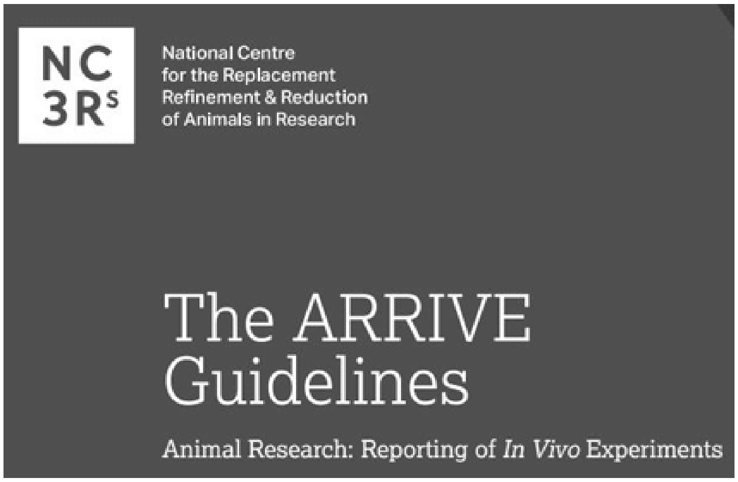

| | ITEM | RECOMMENDATION |
|---|---|---|
| Title | 1 | Provide as accurate and concise a description of the content of the article as possible. |
| Abstract | 2 | Provide an accurate summary of the background, research objectives, including details of the species or strain of animal used, key methods, principal findings and conclusions of the study. |
| INTRODUCTION | | |
| Background | 3 | a. Include sufficient scientific background (including relevant references to previous work) to understand the motivation and context for the study, and explain the experimental approach and rationale.<br><br>b. Explain how and why the animal species and model being used can address the scientific objectives and, where appropriate, the study's relevance to human biology. |
| Objectives | 4 | Clearly describe the primary and any secondary objectives of the study, or specific hypotheses being tested. |
| METHODS | | |
| Ethical statement | 5 | Indicate the nature of the ethical review permissions, relevant licences (e.g. Animal [Scientific Procedures] Act 1986), and national or institutional guidelines for the care and use of animals, that cover the research. |
| Study design | 6 | For each experiment, give brief details of the study design including:<br><br>a. The number of experimental and control groups.<br><br>b. Any steps taken to minimise the effects of subjective bias when allocating animals to treatment (e.g. randomisation procedure) and when assessing results (e.g. if done, describe who was blinded and when).<br><br>c. The experimental unit (e.g. a single animal, group or cage of animals).<br><br>A time-line diagram or flow chart can be useful to illustrate how complex study designs were carried out. |
| Experimental procedures | 7 | For each experiment and each experimental group, including controls, provide precise details of all procedures carried out.<br><br>For example:<br>a. How (e.g. drug formulation and dose, site and route of administration, anaesthesia and analgesia used [including monitoring], surgical procedure, method of euthanasia). Provide details of any specialist equipment used, including supplier(s).<br><br>b. When (e.g. time of day).<br><br>c. Where (e.g. home cage, laboratory, water maze).<br><br>d. Why (e.g. rationale for choice of specific anaesthetic, route of administration, drug dose used). |
| Experimental animals | 8 | a. Provide details of the animals used, including species, strain, sex, developmental stage (e.g. mean or median age plus age range) and weight (e.g. mean or median weight plus weight range).<br><br>b. Provide further relevant information such as the source of animals, international strain nomenclature, genetic modification status (e.g. knock-out or transgenic), genotype, health/immune status, drug or test naïve, previous procedures, etc. |

| | | |
|---|---|---|
| Housing and husbandry | 9 | Provide details of:<br><br>a. Housing (type of facility e.g. specific pathogen free [SPF]; type of cage or housing; bedding material; number of cage companions; tank shape and material etc. for fish).<br><br>b. Husbandry conditions (e.g. breeding programme, light/dark cycle, temperature, quality of water etc for fish, type of food, access to food and water, environmental enrichment).<br><br>c. Welfare-related assessments and interventions that were carried out prior to, during, or after the experiment. |
| Sample size | 10 | a. Specify the total number of animals used in each experiment, and the number of animals in each experimental group.<br><br>b. Explain how the number of animals was arrived at. Provide details of any sample size calculation used.<br><br>c. Indicate the number of independent replications of each experiment, if relevant. |
| Allocating animals to experimental groups | 11 | a. Give full details of how animals were allocated to experimental groups, including randomisation or matching if done.<br><br>b. Describe the order in which the animals in the different experimental groups were treated and assessed. |
| Experimental outcomes | 12 | Clearly define the primary and secondary experimental outcomes assessed (e.g. cell death, molecular markers, behavioural changes). |
| Statistical methods | 13 | a. Provide details of the statistical methods used for each analysis.<br><br>b. Specify the unit of analysis for each dataset (e.g. single animal, group of animals, single neuron).<br><br>c. Describe any methods used to assess whether the data met the assumptions of the statistical approach. |
| RESULTS | | |
| Baseline data | 14 | For each experimental group, report relevant characteristics and health status of animals (e.g. weight, microbiological status, and drug or test naïve) prior to treatment or testing (this information can often be tabulated). |
| Numbers analysed | 15 | a. Report the number of animals in each group included in each analysis. Report absolute numbers (e.g. 10/20, not 50%[2]).<br><br>b. If any animals or data were not included in the analysis, explain why. |
| Outcomes and estimation | 16 | Report the results for each analysis carried out, with a measure of precision (e.g. standard error or confidence interval). |
| Adverse events | 17 | a. Give details of all important adverse events in each experimental group.<br><br>b. Describe any modifications to the experimental protocols made to reduce adverse events. |
| DISCUSSION | | |
| Interpretation/ scientific implications | 18 | a. Interpret the results, taking into account the study objectives and hypotheses, current theory and other relevant studies in the literature.<br><br>b. Comment on the study limitations including any potential sources of bias, any limitations of the animal model, and the imprecision associated with the results[2].<br><br>c. Describe any implications of your experimental methods or findings for the replacement, refinement or reduction (the 3Rs) of the use of animals in research. |
| Generalisability/ translation | 19 | Comment on whether, and how, the findings of this study are likely to translate to other species or systems, including any relevance to human biology. |
| Funding | 20 | List all funding sources (including grant number) and the role of the funder(s) in the study. |

* 18. How appropriate is it to provide details on analgesia, anesthesia, antibiotics, antiseptic measures, intestinal segment involved, surgical technique and anastomotic complications in animal research?

| 1: Very inappropriate | 2 | 3 | 4 | 5 | 6 | 7 | 8 | 9: Very appropriate |
|---|---|---|---|---|---|---|---|---|
| ○ | ○ | ○ | ○ | ○ | ○ | ○ | ○ | ○ |

Why is it considered (in)appropriate/additional remarks

* 19. How appropriate is it to provide information on randomization and blinding methods in animal research?

| 1: Very inappropriate | 2 | 3 | 4 | 5 | 6 | 7 | 8 | 9: Very appropriate |
|---|---|---|---|---|---|---|---|---|
| ○ | ○ | ○ | ○ | ○ | ○ | ○ | ○ | ○ |

Why is it considered (in)appropriate/additional remarks

* 20. How appropriate would an online registration of the protocol (like clinicaltrials.gov for patient trials) be for animal research?

| 1: Very inappropriate | 2 | 3 | 4 | 5 | 6 | 7 | 8 | 9: Very appropriate |
|---|---|---|---|---|---|---|---|---|
| ○ | ○ | ○ | ○ | ○ | ○ | ○ | ○ | ○ |

Why is it considered (in)appropriate/additional remarks

Maastricht UMC+

azM | Maastricht University

## Final Round: The use of animal models for research on anastomoses in the lower gastrointestinal tract

### Thank you very much!

**We like to thank you for completing this final round of the Delphi survey. All responses will be analysed and the results will be reported back to you in the form of recommendations for animal research on bowel anastomoses in the lower gastrointestinal tract.**
**Despite a previous announcement about a potential conference call or meeting, due to the international character of the panel, we were advised to complete the Delphi analysis online.**
**If you have any remarks at this point, please feel free to comment in the box below.**

**If you want direct contact through email, please send your message to:**
**n.bouvy@mumc.nl or ac.bosmans@maastrichtuniversity.nl**

**Thank you very much for participating in this international consensus project!**

21. Additional remarks
